# The complete chloroplast genome sequence of *Ormosia formosana*

**DOI:** 10.1080/23802359.2020.1782278

**Published:** 2020-07-06

**Authors:** Zheng-Feng Wang, Li-Wan Chang, Ju-Yu Lian, Hong-Lin Cao

**Affiliations:** a Key Laboratory of Vegetation Restoration and Management of Degraded Ecosystems, South China Botanical Garden, Chinese Academy of Sciences, Guangzhou; b Center for Plant Ecology, Core Botanical Gardens, Chinese Academy of Sciences, Guangzhou; c Southern Marine Science and Engineering Guangdong Laboratory (Guangzhou), Guangzhou; d Forest Protection Division, Taiwan Forestry Research Institute, Taipei

**Keywords:** Chloroplast, genome assembly, high-throughput sequencing, *Ormosia formosana*

## Abstract

*Ormosia formosana* is an important hardwood species and its seeds are popular as decorative jewelry. Currently, this species is threatened in the natural forests due to habitat destruction. Here, we first report the chloroplast genome of *O. formosana* for future studies in ecology, phylogeny, and conservation. The chloroplast genome of *O. formosana* is 173,587 bp in length with a GC content of 35.80%. It includes a large single-copy region of 73,550 bp, a small single-copy region of 18,683 bp, and two inverted repeat regions of 40,696 bp and 40,658 bp, respectively. The genome was totally annotated with 135 genes, including 90 protein-coding genes, eight ribosomal RNA genes, and 37 transfer RNA genes. Phylogenetic analysis indicated that *O. formosana* is most genetically similar to *O. boluoensis*.

*Ormosia formosana* belongs to the Fabaceae family. It naturally grows in evergreen broad-leaved forests at elevations between 300 and 1000 m on Taiwan Island. *O. formosana* is an important hardwood species with trees that can grow to a height of 20 m and with trunks ranging in diameter from 17 to 50 cm when mature. The wood from these trees is hard and heavy, with a reddish color and somewhat coarse grain, and is used for construction and furniture. *O. formosana* produces striking red colored seeds that are used for jewelry, beads and other decorative purposes. These trees are also known to be the only natural host plant for the *Hasora anura* de Nicéville butterfly in this region (Hsu et al. 2005). However, due to habitat destruction, *O. formosana* is threatened in the natural forests. Therefore, in this study, we report the complete chloroplast genomic sequence of *O. formosana* in order to provide a useful genetic resource for future effective conservation.

Plant materials were sampled from the Lienhuachih Forest Dynamics Plot (23°54′49″N, 120°52′43″E) on Taiwan Island and a voucher specimen was deposited in the Herbarium of Taiwan Forest Research Institute with No. 524800. The chloroplast genome of *O. formosana* was assembled by NOVOPlasty 3.7.2 (Dierckxsens et al. 2017) using whole genome sequencing reads (about 110 Gb) generated on the Illumina HiSeq X Ten (Illumina, San Diego, CA, USA) platform using the paired-end (2 × 150 bp) strategy. The assembled chloroplast genome was then gene annotated using PGA (Qu et al. 2019) and deposited in GenBank with the accession number MT258921. To perform phylogenetic analysis, 70 protein-coding genes were extracted from the chloroplast genome of *O. formosana*, as well as nine other Papilionoideae species, using PhyloSuite 1.2.1 (Zhang et al. 2020). Subsequently, these genes were concatenated and used for Bayesian phylogenetic inference with MrBayes 3.2.7a (Huelsenbeck and Ronquist 2001). The nine chloroplast genomes used were *O. boluoensis* (GenBank accession No. MN886968), *O. emarginata* (No. MK105448.1), *O. hosiei* (No. MG813874.1), *O. semicastrata* (No. MK105450.1), *O. xylocarpa* (No. MK105449.1), *Salweenia bouffordiana* (No. MF449303.1), *Sophora alopecuroides* (No. NC036102.1), *Sophora flacescens* (No. MH748034.1), and *Sophora tonkinensis* (No. NC042688.1).

The length of the complete chloroplast genome of *O. formosana* was 173,587 bp. The GC content of the genome was 35.80%. The genome sequence contained a large single-copy region of 73,550 bp, a small single-copy region of 18,683 bp, and two inverted repeat (IRA and IRB) regions of 40,696 bp and 40,658 bp, respectively. The genome consisted of a total of 135 genes, including 90 protein-coding genes, eight ribosomal RNA genes, and 37 transfer RNA genes. The phylogenetic tree demonstrated that *O. formosana* is most closely evolutionarily related to *O. boluoensis*, with strong support (Figure 1).

**Figure 1.**
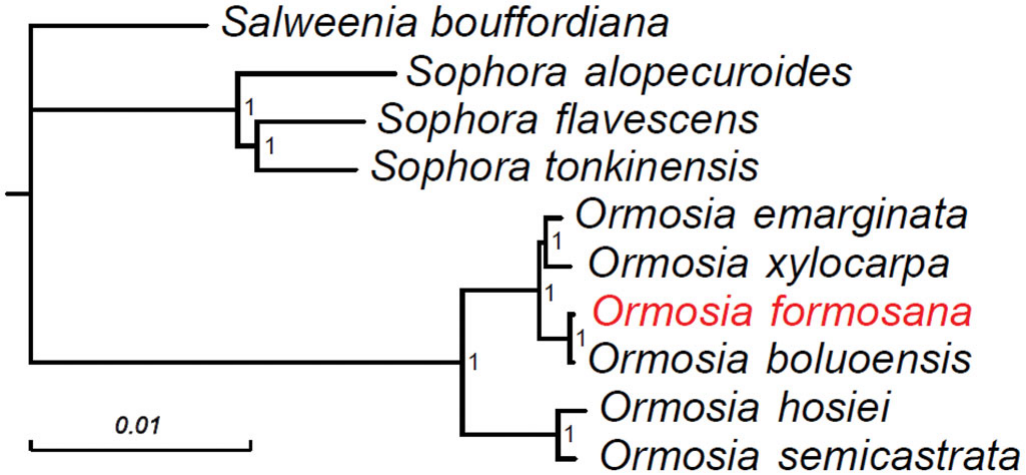
Phylogenetic tree constructed using 70 protein-coding genes from the complete chloroplast genome sequences of six *Ormosia* species and four other species within the Papilionoideae. The numbers beside the node indicate Bayesian posterior probability.

## Data Availability

The complete chloroplast genome sequences of *Ormosia formosana* have been deposited in GenBank under the accession numbers MT258921 and are accessible at https://doi.org/10.13140/RG.2.2.27661.90087.
